# QMODE+ ablation mode: optimal parameter setting and impedance-adapted strategies

**DOI:** 10.3389/fcvm.2026.1743828

**Published:** 2026-02-05

**Authors:** Xuesong Shi, Mingyu Sun, Zulu Wang, Ming Liang, Zhiqing Jin, Jian Ding

**Affiliations:** 1 State Key Laboratory of Frigid Zone Cardiovascular Disease, Cardiovascular Research Institute and Department of Cardiology, General Hospital of Northern Theater Command, Shenyang, China; 2 Dalian Medical University, Dalian, China

**Keywords:** impedance, impedance-adapted strategies, lesion characteristics, parameter setting, QMODE+ ablation

## Abstract

**Background:**

A new catheter (the QDOT-MICRO catheter) is used for high-power short duration (vHPSD) ablation.

**Objectives:**

This study aimed to optimize lesion formation in QMODE+ ablation (using QDOT-MICRO catheter) by clarifying effects of key parameters and developing impedance-adapted strategies.

**Methods:**

Radio frequency (RF) ablation was performed on excised porcine myocardium with QDOT-MICRO catheter under QMODE+ ablation mode. Parameters included contact force (CF), power, RF duration, interlesion distance, contact angle, and impedance. The optimal parameter was determined, and adjustments for consistent lesion formation across varying impedances were investigated.

**Results:**

In QMODE+ ablation mode, lesion size (surface width and depth) increased with higher CF, longer RF duration, and greater power, but decreased as impedance elevated. However, lesions became smaller when CF exceeded 25 g, due to rapid temperature drops from the QMODE+ temperature control system. Perpendicular contact resulted in greater lesion depth compared to parallel contact. An interlesion distance of 5 mm was used as an experimentally evaluated reference condition and produced continuous lesions without excessive overlap. For impedance adjustments, 80 W/3 s, 80 W/4 s, and 90 W/3 s at 90Ω under QMODE+ ablation mode produced lesions comparable to those at 120Ω; when the impedance was high at 150Ω, switching to QMODE ablation mode with AI = 400/420 achieved similar lesion characteristics comparable to those at 120Ω.

**Conclusions:**

Routine QMODE+ ablation with 90 W power, 4 s RF duration, catheter CF < 25 g, 5 mm interlesion distance (reference in this *ex vivo* model), and perpendicular contact achieves optimal lesion formation. To ensure consistent damage across different impedances, adjust QMODE+ parameters at low impedance and switch to QMODE at high impedance. Parallel contact could be preferred at fragile sites or under low impedance to reduce lesion depth and avoid over-damage.

## 1 Introduction

Although pulsed field ablation has gained significant traction in electrophysiology, radio frequency (RF) energy remains a widely used modality for pulmonary vein isolation (PVI) of atrial fibrillation (AF). Establishing durable RF lesions is critical to reducing arrhythmia recurrence (1, 2). Due to technical and procedural optimization in RF ablation, long-term success rates for PVI in patients with paroxysmal atrial fibrillation (PAF) are satisfying. Nevertheless, the main cause for AF recurrence is related to pulmonary vein (PV) reconnection due to initially non-transmural lesions and tissue oedema (3, 4). Factors such as ablation power, duration, catheter-tissue contact force (CF), and impedance significantly influence lesion formation (5–8). High power and short duration (HPSD; ≥50 W, typically <15 s) radio frequency ablation replacement strategies have been widely used in clinical settings to maintain or even improve the effectiveness and safety of lesion creation (9). Clinical applications of HPSD are limited to 50 W. Recently, a catheter with a multi-thermocouple system has been developed for accurate temperature monitoring, allowing real-time temperature assessment of the catheter-tissue interface (10–13). This new catheter is capable of performing extremely very high power short duration (vHPSD) applications (up to 90 W and 4 s) in QMODE+ ablation mode. Current research on vHPSD ablation parameters remains limited, with no systematic studies specifically addressing this field, particularly the critical issue of parameter adjustment strategies under varying impedance conditions. Therefore, the objective of this study was to systematically characterize lesion geometry under vHPSD ablation in an *ex vivo* myocardial model, focusing on the effects of power, impedance, catheter orientation, and inter-lesion spacing. We further evaluated optimal parameter adjustment strategies across different impedance scenarios.

## 2 Methods

### 2.1 *Ex vivo* experimental models

Freshly excised porcine hearts, obtained within 12–48 h, were used in the experiments. The cardiac tissues were obtained from a licensed commercial slaughterhouse in Shenyang, Liaoning Province, China, as food-industry by-products following routine slaughter for human consumption. The hearts were placed in a circulating saline bath (400 mL/min flow rate, 3.5 g/L NaCl). The atrium was dissected into uniform slices and submerged in a 37 ℃ saline bath. Details of this *in vitro* model have been previously described (14, 15). A 3.5 mm tip-irrigated ablation catheter QDOT MICRO™, Biosense Webster, Irvine, CA, USA), featuring three microelectrodes and six thermocouples, was utilized for the RF applications. The catheter was securely fixed in a plastic tube within the tank to ensure stable RF power delivery during ablation.

### 2.2 Ablation settings

All RF applications were delivered using a point-by-point ablation technique. Dragging or continuous ablation was not performed in any experimental condition. During energy delivery, the ablation catheter was maintained in a stable position with a near-perpendicular orientation to the myocardial surface. Catheter orientation and contact force were kept constant throughout each application to minimize variability related to catheter–tissue interaction. The vHPSD was performed at 90 W for 4 s using the QDOT-MICRO catheter, whereas our study focused on adjusting these parameters within QMODE+ ablation mode—specifically modifying CFs, power, ablation duration, interlesion distance, contact angle and impedance. Controlling for a single variable, we examined the changes in lesion characteristics. To ensure consistent ablation damage across varying impedances, impedance categories were established, with corresponding adjustments to power and duration to achieve comparable lesion formation. For persistent inconsistencies despite QMODE+ optimization, QMODE ablation mode was adopted as an alternative to refine energy delivery. During interlesion distance experiments, the catheter was kept at a constant contact angle and orientation: the catheter long axis was oriented perpendicular to the intended ablation line, and successive applications were delivered in the same direction, while contact force was maintained within the preset range (10–15 g) for that experiment. Interlesion distances of ≥3 mm were predefined and tested as experimental conditions. These distances were selected to represent commonly used spacing ranges in ablation and to allow controlled comparison of lesion continuity in an *ex vivo* setting. For the additional QMODE ablation mode experiments at 150Ω, power was fixed at 50 W with CF maintained at 10–15 g, and RF delivery was terminated when the preset AI target (360–460) was reached.

### 2.3 RF lesion assessment

The lesion's long axis was crossed, and the white area was measured using a digital caliper with a resolution of 0.01 mm to determine both the maximum depth and the maximum surface diameter of the lesion. All lesion measurements were performed by the same individual, who was blinded to the ablation setting parameters during the measurement process. To investigate ablation inconsistencies under varying impedances, 2 mm-thick frozen sections were prepared (16, 17). The sections were placed in a 1% triphenyl tetrazolium chloride (TTC) solution, immersed in TTC-containing dishes, and incubated in a 37 °C light-protected water bath for 15–30 min with continuous rotation to ensure uniformity. TTC induced red coloration of mitochondrial succinate dehydrogenase in viable cells, distinguishing live (red) from non-viable (pale) tissues (16, 18). Following reaction termination, the sections were washed with PBS and fixed with 4% polyformaldehyde. Steam pops were monitored continuously by direct visual inspection, audible cues, and real-time impedance tracing on the generator.

### 2.4 Statistical analysis

Continuous variables, including lesion surface width and lesion depth, were expressed as mean ± standard deviation (SD). Comparisons among multiple experimental groups were performed using one-way analysis of variance (ANOVA). Due to the multiple comparisons involving lesion features, pairwise comparisons were adjusted using the Bonferroni correction to assess significant differences. Categorical variables were presented as counts (percentages) and compared using the *χ*^2^ test or Fisher's exact test, as appropriate. A *p*-value of <0.05 was considered statistically significant. All statistical analyses were performed using SPSS 27.0 software.

## 3 Results

Across all applications, no steam pops were observed under the monitoring methods described above.

### 3.1 Lesion characteristics in QMODE+ ablation mode using different CFs

**Figure 1:**
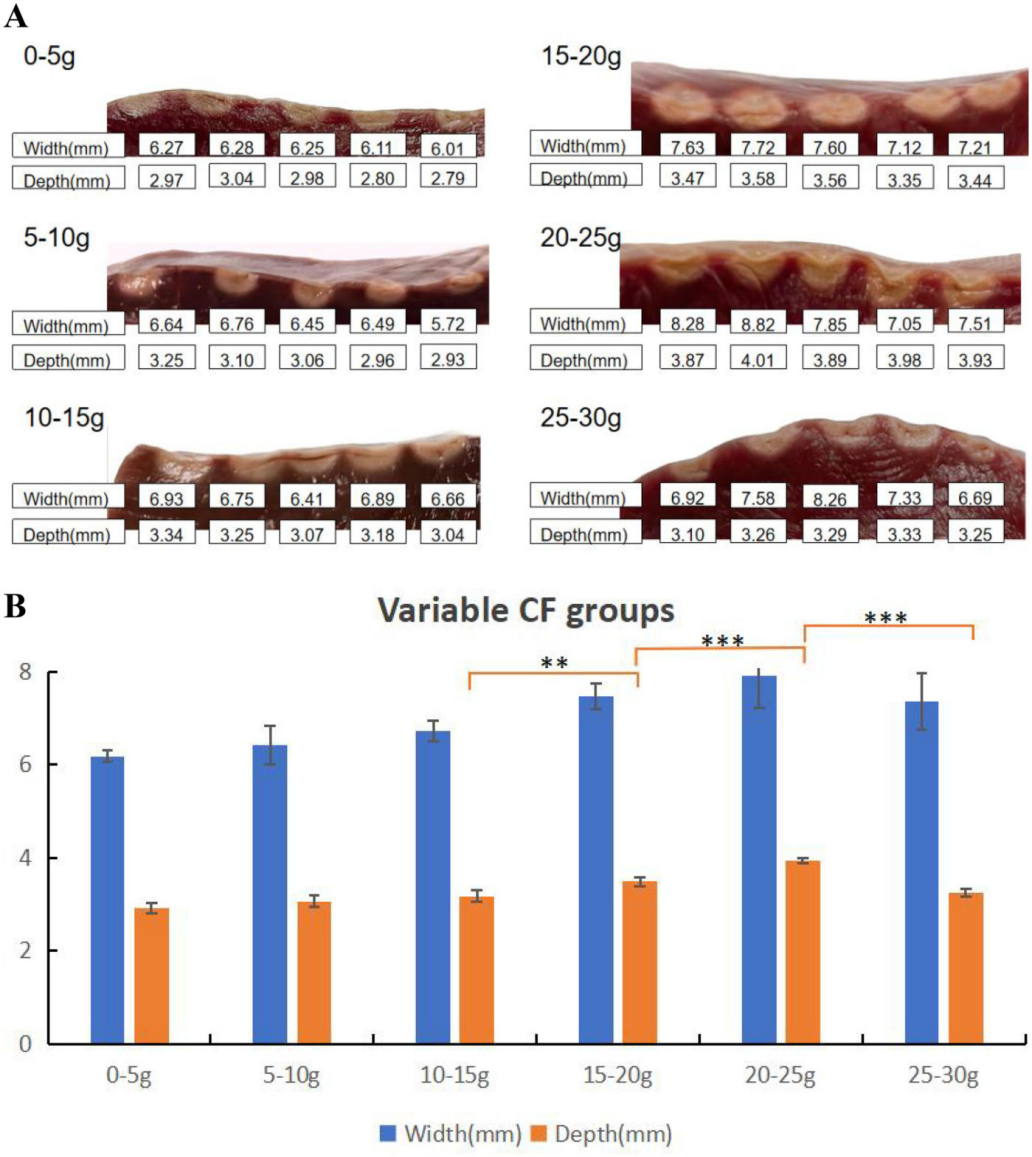
Effect of CFs on ablation lesion extent. **(A)** Gross lesion morphology. **(B)** Surface width and depth of lesions formed when the catheter was in perpendicular contact with tissue in QMODE+ ablation mode. Statistical significance is indicated as *P < 0.05, **P < 0.01, ***P < 0.001.

**Figure 2:**
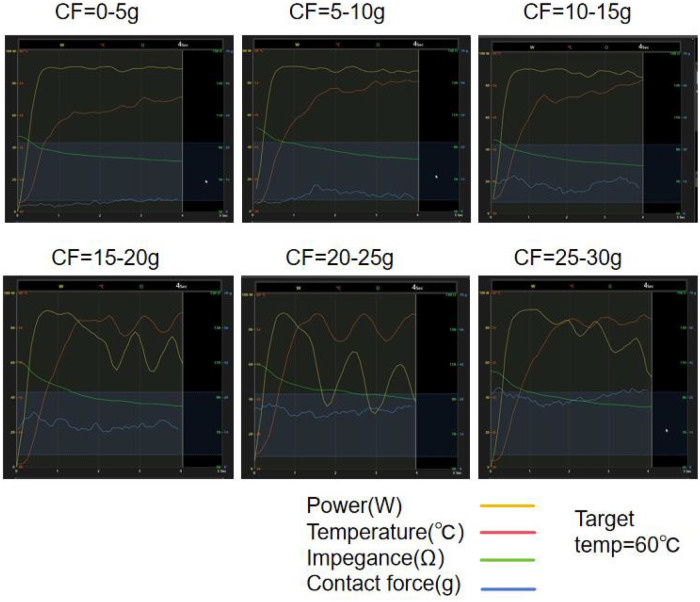
An example of the QMODE+ ablation parameters created by RF application with a target temperature of 60°C under varying CF is shown. When CF exceeds 15 g, frequent power titration occurs, and the temperature drops rapidly.

The CF groups were set as follows: 0–5 g, 5–10 g, 10–15 g, 15–20 g, 20–25 g, and 25–30 g, with five lesions in each group (see Table 1). Lesion characteristics were illustrated in Figure 1A. The smallest lesion depth and surface width were observed in the 0–5 g CF group. As shown in Table 1, lesion depth and surface width increased with increasing CF in the range < 25 g. However, when CF reached over 25 g, lesion depth and surface width did not increase further; instead, they showed signs of reduced damage. When comparing adjacent groups, no statistical difference was observed in surface width, but significant differences in lesion depth emerged when CF > 10–15 g (*P* < 0.01, *P* < 0.001, *P* < 0.001) (as shown in Figure 1B). Figure 2 presents examples of QMODE+ ablation mode parameters during RF application at a target temperature of 60℃ under different CF conditions: when CF exceeded 15 g, the temperature drop intensified, particularly over 25 g, resulting in relatively smaller lesions.

**Table 1:** Lesion characteristics in QMODE+ ablation mode using different CFs.

Lesion Parameters	CF = 0–5 g	CF = 5–10 g	CF = 10–15 g	CF = 15–20 g	CF = 20–25 g	CF = 25–30 g
	*N* = 5	*N* = 5	*N* = 5	*N* = 5	*N* = 5	*N* = 5
Surface width	6.18 ± 0.12	6.41 ± 0.41	6.72 ± 0.21	7.46 ± 0.27	7.90 ± 0.68	7.36 ± 0.61
Lesion depth	2.92 ± 0.11	3.06 ± 0.13	3.17 ± 0.12	3.48 ± 0.09	3.94 ± 0.06	3.25 ± 0.09

CF, contact force.

### 3.2 Lesion characteristics in QMODE+ ablation mode using different power

**Figure 3:**
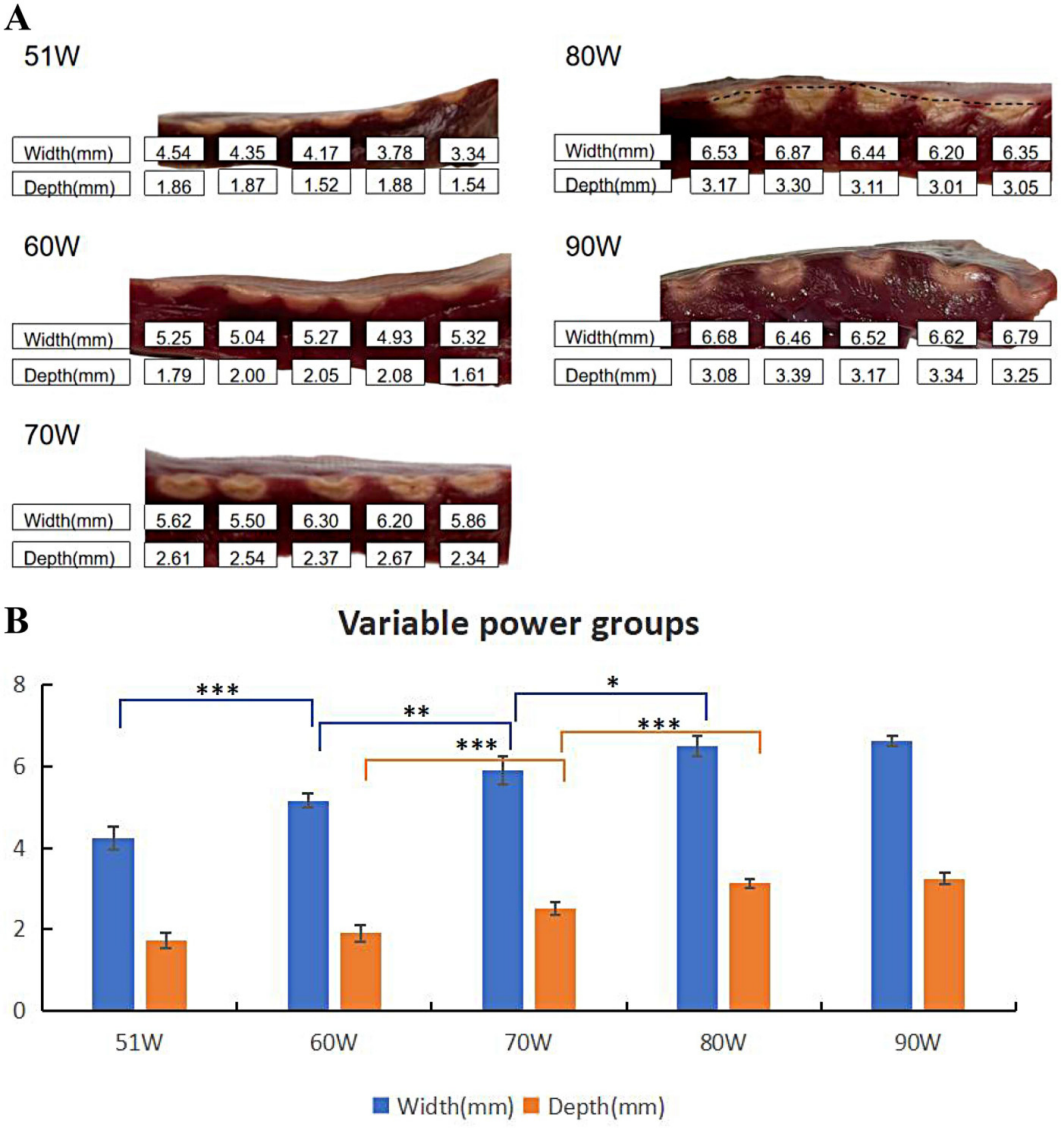
Effect of power on ablation lesion extent. **(A)** Gross lesion morphology. **(B)** Surface width and depth of lesions formed using different power when the catheter was in perpendicular contact with tissue in QMODE+ ablation mode. Statistical significance is indicated as *P < 0.05, **P < 0.01, ***P < 0.001.

Different power (90 W, 80 W, 70 W, 60 W, and 51 W-the minimum procedure setting) were used to generate 5 lesions in each group (Table 2). Both the surface width and lesion depth were greatest at 90 W, and decreased as the power wattage decreased. When comparing adjacent groups, there was a statistical difference in surface width between the 51 W vs. 60 W, 60 W vs. 70 W, and 70 W vs. 80 W groups (*P* < 0.001, *P* = 0.002, *P* = 0.015), and a statistical difference in lesion depth between the 60 W vs. 70 W and 70 W vs. 80 W groups (all *P* < 0.001) (as shown in Figure 3B). The lesion features are shown in Figure 3A.

**Table 2:** Lesion characteristics in QMODE+ ablation mode using different power.

Lesion Parameters	51 W	60 W	70 W	80 W	90 W
Surface width	4.24 ± 0.29	5.16 ± 0.17	5.90 ± 0.35	6.48 ± 0.25	6.61 ± 0.13
Lesion depth	1.73 ± 0.19	1.91 ± 0.20	2.51 ± 0.15	3.13 ± 0.11	3.25 ± 0.13

*N* = 5 in each group.

### 3.3 Lesion characteristics in QMODE+ ablation mode with different RF durations

**Figure 4:**
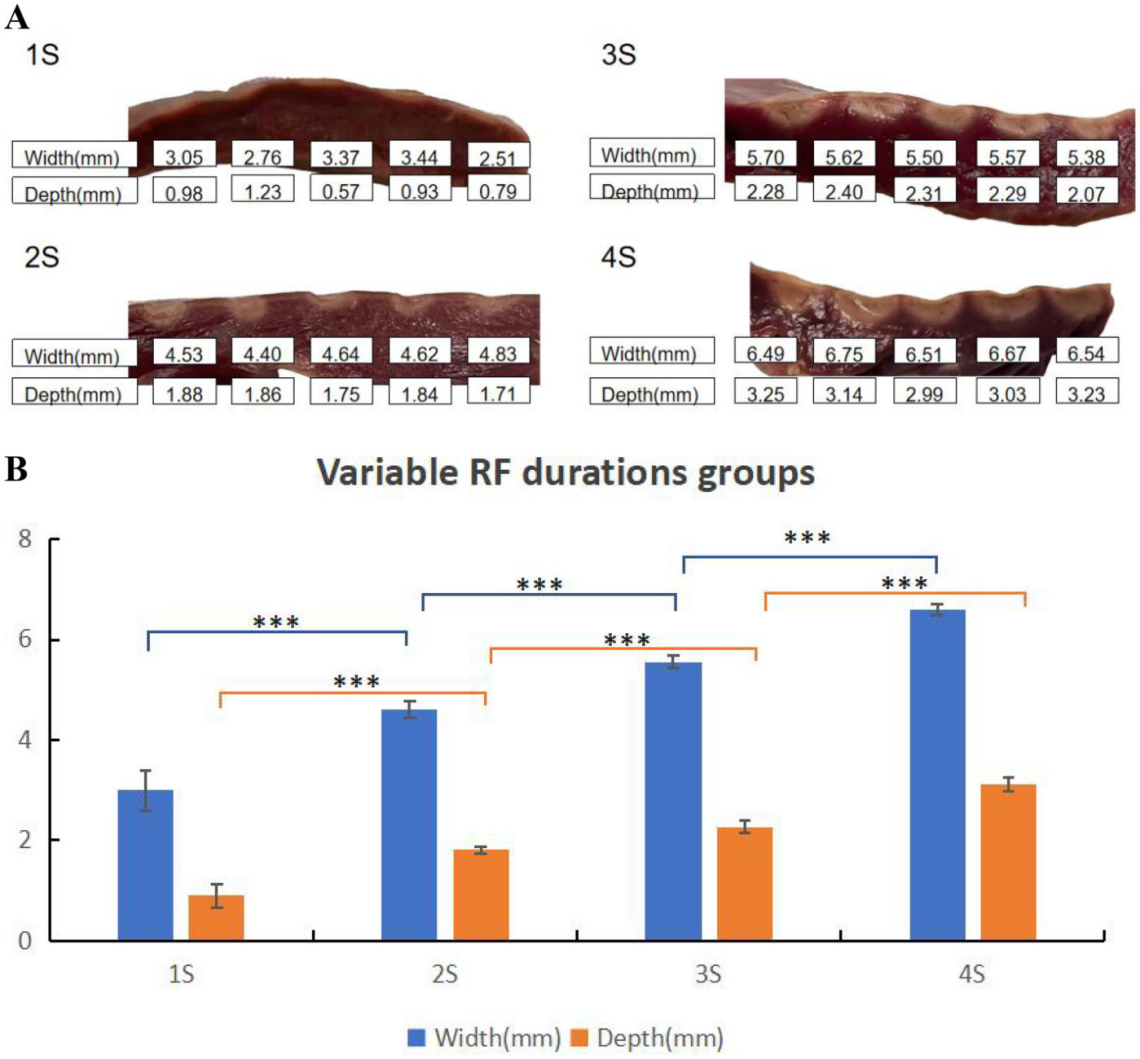
Effect of RF durations on ablation lesion extent. **(A)** Gross lesion morphology. **(B)** Surface width and depth of lesions formed under different RF durations when the catheter was in perpendicular contact with tissue in QMODE+ ablation mode. Statistical significance is indicated as *P < 0.05, **P < 0.01, ***P < 0.001.

The RF durations were divided into four groups: 4 s, 3 s, 2 s, and 1 s, with 5 lesions in each group (as shown in Table 3 and Figure 4A). Mean values indicated that lesion size increased as RF duration prolonged. Statistically significant differences in lesion surface width and depth were observed across all groups (Figure 4B).

**Table 3:** Lesion characteristics in QMODE+ ablation mode with different RF durations.

Lesion Parameters	1 S	2 S	3 S	4 S
Surface width	3.00 ± 0.40	4.60 ± 0.16	5.55 ± 0.12	6.59 ± 0.11
Lesion depth	0.90 ± 0.24	1.81 ± 0.07	2.27 ± 0.12	3.24 ± 0.14

*N* = 5 in each group.

### 3.4 Lesion characteristics in QMODE+ ablation mode at different contact angles

**Figure 5:**
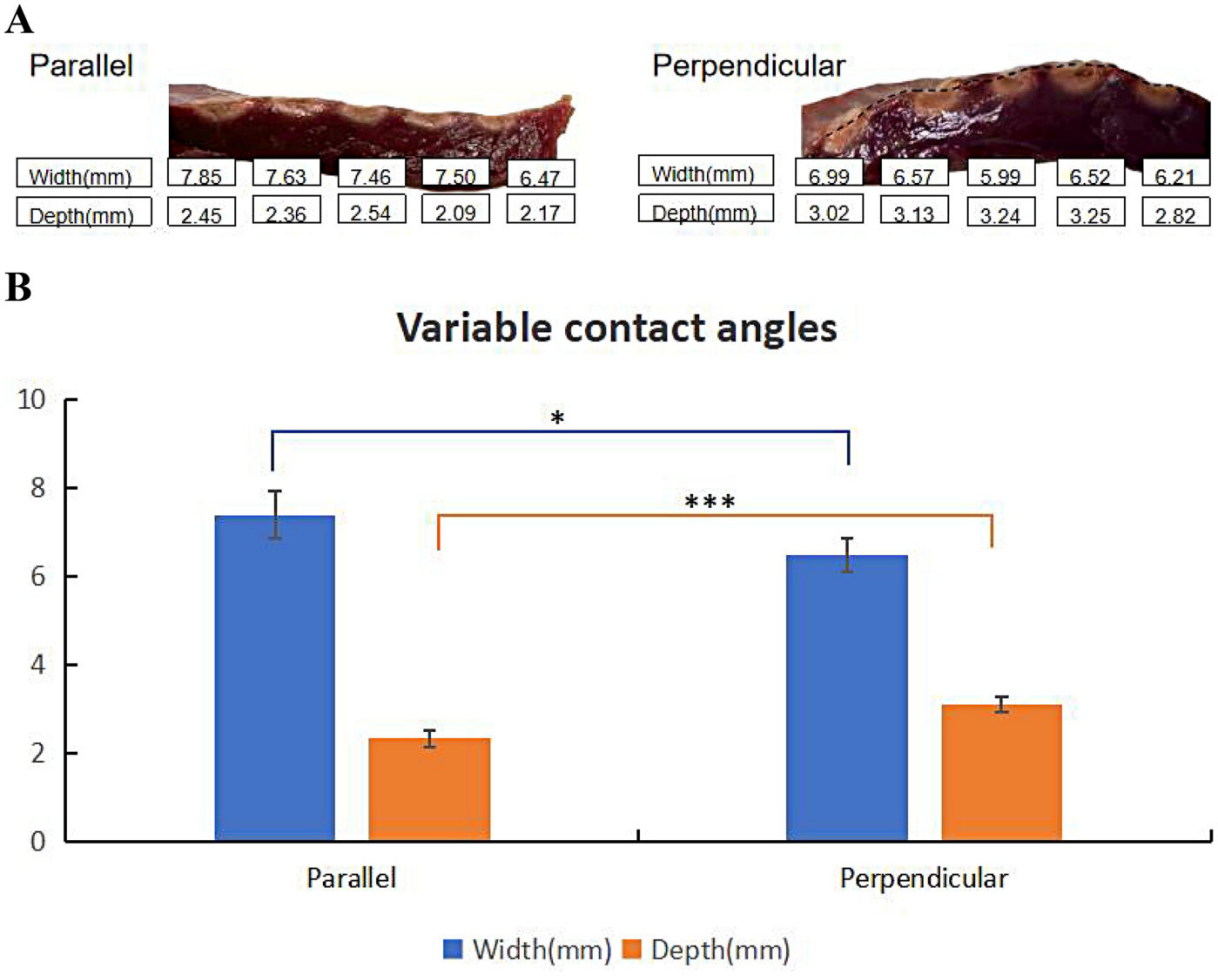
Effect of contact angles on ablation lesion extent. **(A)** Gross lesion morphology. **(B)** Surface width and depth of lesions formed under different contact angles with tissue in QMODE+ ablation mode. Statistical significance is indicated as *P < 0.05, **P < 0.01, ***P < 0.001.

Generally, due to the larger contact area in the parallel contact group, the average surface widths were greater than those in the perpendicular contact group. Conversely, the average tissue depths in the perpendicular contact group exceeded those in the parallel contact group (as shown in Table 4). Figure 5A shows the effect of different contact angles of ablation catheter on lesion formation. Statistically significant differences in lesion surface width and lesion depth were observed across all groups (Figure 5B).

**Table 4:** Lesion characteristics in QMODE+ ablation mode at different contact angles.

Lesion Parameters	Parallel	Perpendicular
Surface width	7.38 ± 0.53	6.46 ± 0.38
Lesion depth	2.32 ± 0.19	3.09 ± 0.18

*N* = 5 in each group.

### 3.5 Evaluation of interlesion distance

**Figure 6:**
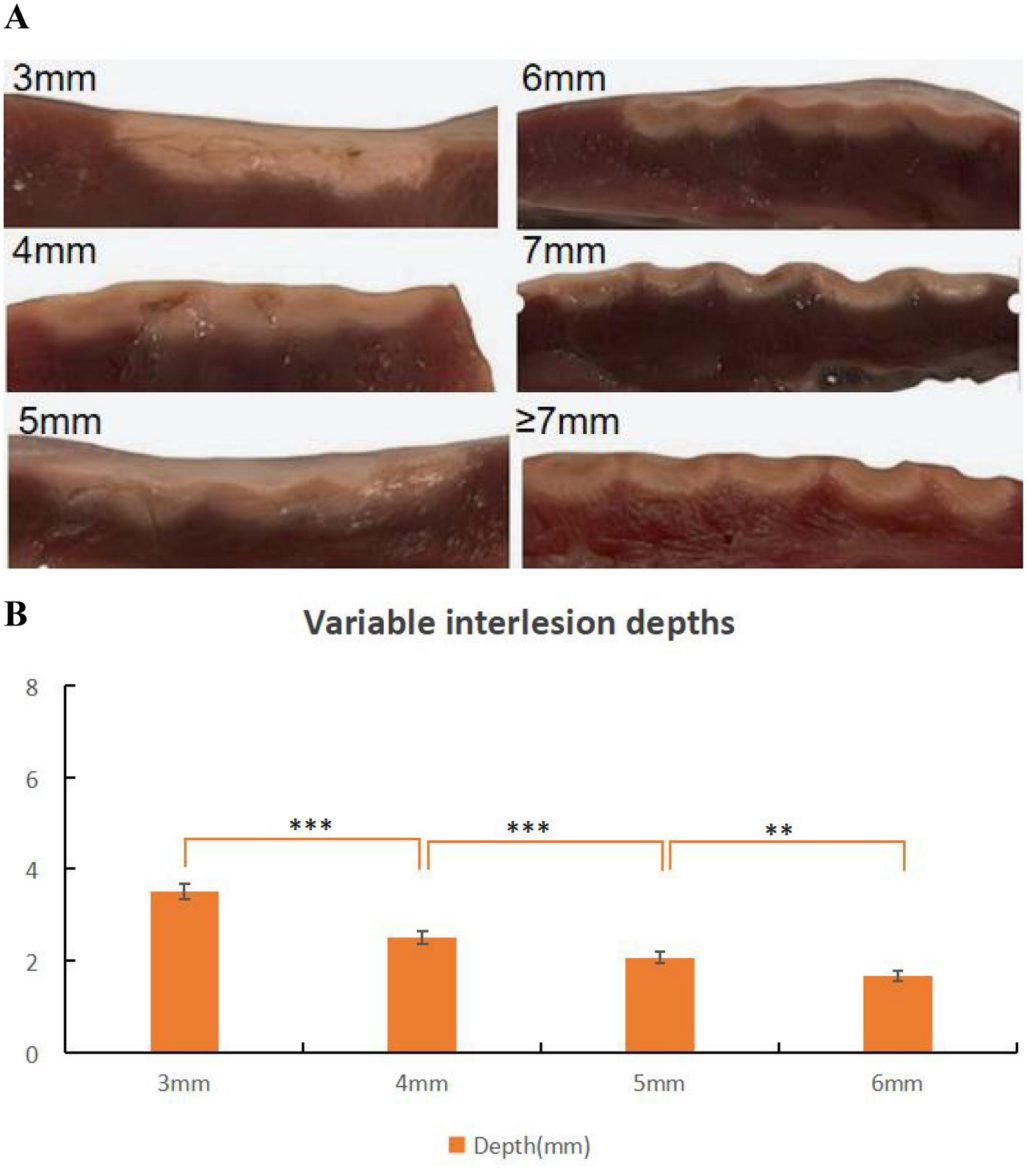
Effect of interlesions on ablation lesion extent. **(A)** Gross lesion morphology. **(B)** Depth of lesions formed under different interlesions with tissue in QMODE+ ablation mode. Statistical significance is indicated as *P < 0.05, **P < 0.01, ***P <v0.001.

The interlesion distance of RF applications was gradually increased, with a minimum distance of 3 mm to a maximum of 7 mm. Ablation interlesion distances at 3 mm and 4 mm significantly increased lesion interlesion depth, whereas a 6 mm interlesion distance resulted in insufficient depth at the connected region, with potentiated possibility of gaps forming (Table 5 and Figure 6B). When the interlesion distance was ≥7 mm, RF applications produced lesions with isolated discontinuities, as shown in Figure 6A.

**Table 5:** Lesion interlesion depth in QMODE+ ablation mode with different interlesion distance.

Lesion Parameters	3 mm	4 mm	5 mm	6 mm
Lesion depth	3.50 ± 0.17	2.51 ± 0.14	2.07 ± 0.12	1.67 ± 0.11

*N* = 5 in each group.

### 3.6 Lesion characteristics in QMODE+ ablation mode with different impedances

**Figure 7:**
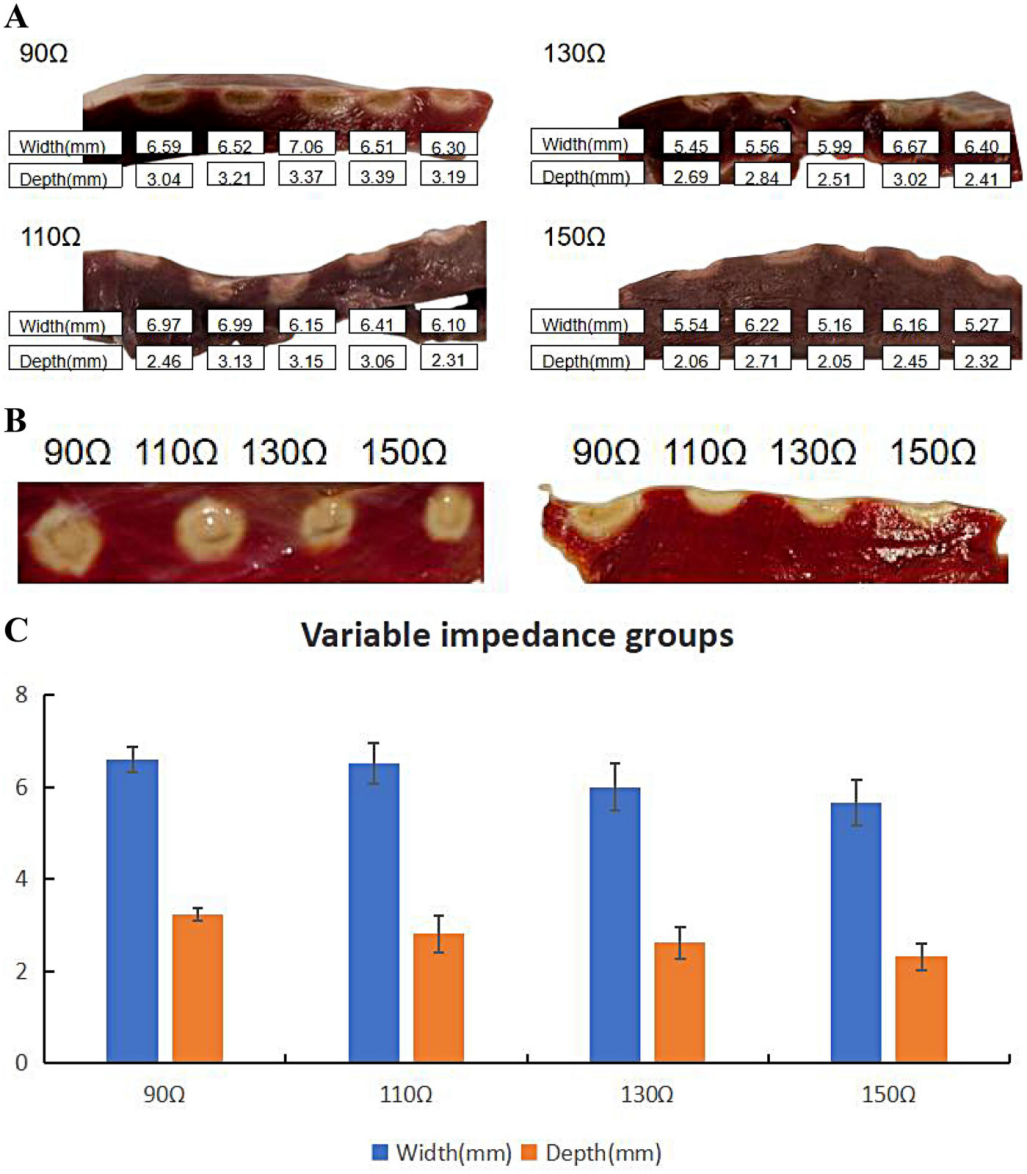
Effect of impedance on ablation lesion extent. **(A)** Gross lesion morphology. **(B)** Comparison of ablation lesions with different impedances in the same TTC-stained myocardium. **(C)** Surface width and depth of lesions formed under different impedances when the catheter was in perpendicular contact with tissue in QMODE+ ablation mode. Statistical significance is indicated as *P < 0.05, **P < 0.01, ***P < 0.001.

Basic impedance was controlled by adjusting saline concentration and electrode plate distance, with four groups established as shown in Table 6 (5 lesions per group). Figure 7A showed representative lesions and three distinct regions were observed in the ablation lesions at 90Ω. Thus, in myocardial specimens stained with TTC, 90Ω lesions were clearly distinguishable to the naked eye. As shown in Figure 7B, TTC staining well demarcated ablation lesions into three distinct regions: (1) a central dark brown area, (2) a lighter-edged region, and (3) normal red myocardial tissue. At a basic impedance of 90Ω, lesions exhibited the maximum average surface width and tissue depth. Lesion extent decreased as basic impedance elevated. No statistical differences in surface width or tissue depth were observed between adjacent groups (Figure 7C).

**Table 6:** Lesion characteristics with different impedances.

Lesion Parameters	90Ω	110Ω	130Ω	150Ω
Surface width	6.60 ± 0.28	6.52 ± 0.43	6.01 ± 0.52	5.67 ± 0.49
Lesion depth	3.24 ± 0.14	2.82 ± 0.40	2.62 ± 0.35	2.32 ± 0.28

*N* = 5 in each group.

### 3.7 Parameter adjustments for consistent lesion formation across varying impedances

**Figure 8:**
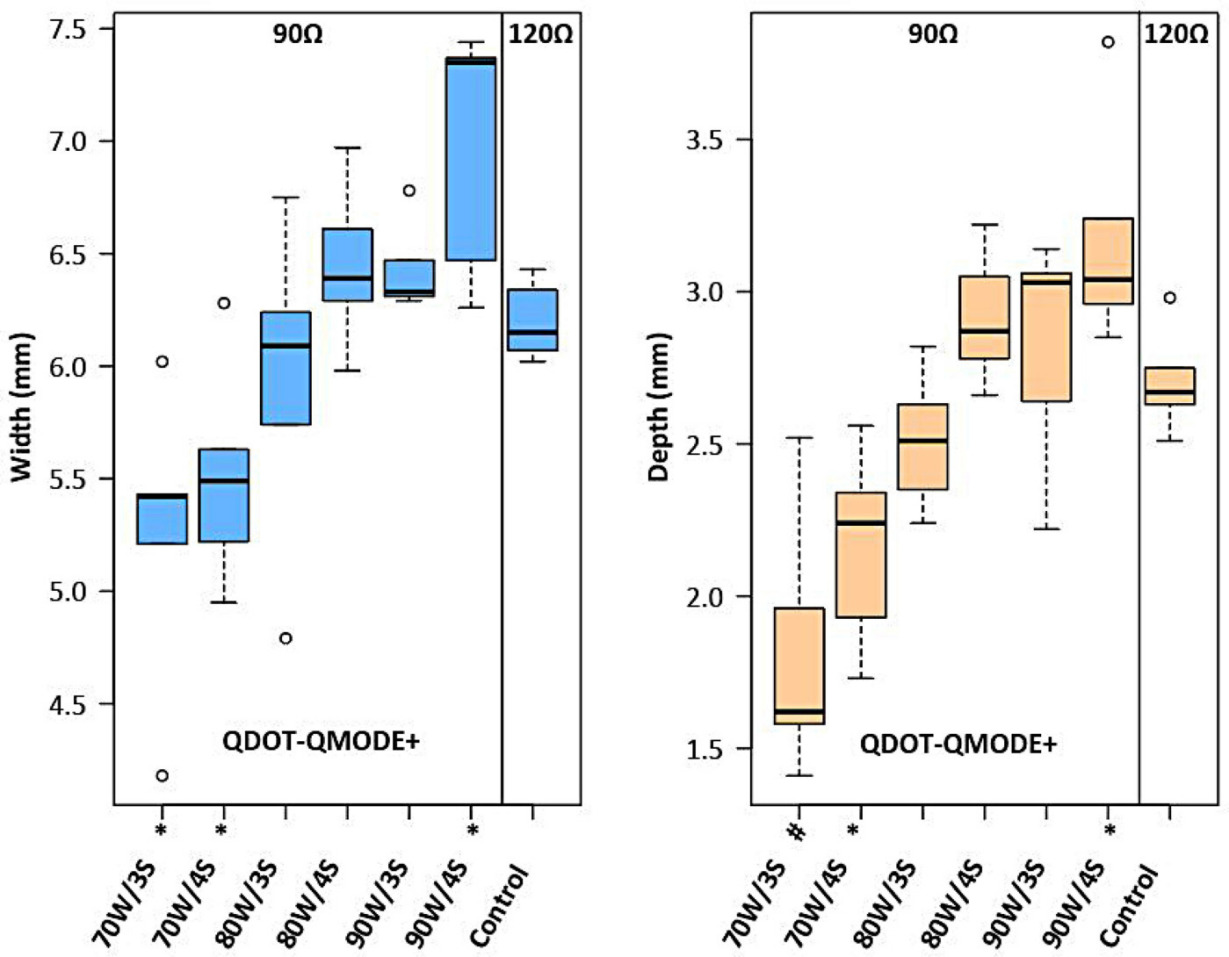
Box plots showing lesion width and depth for each tested ablation setting in 90Ω in QMODE+ ablation mode, compared to the control group (120Ω). Statistical significance is indicated as follows: *P < 0.05, #*P* < 0.01, †*P* < 0.001.

**Figure 9:**
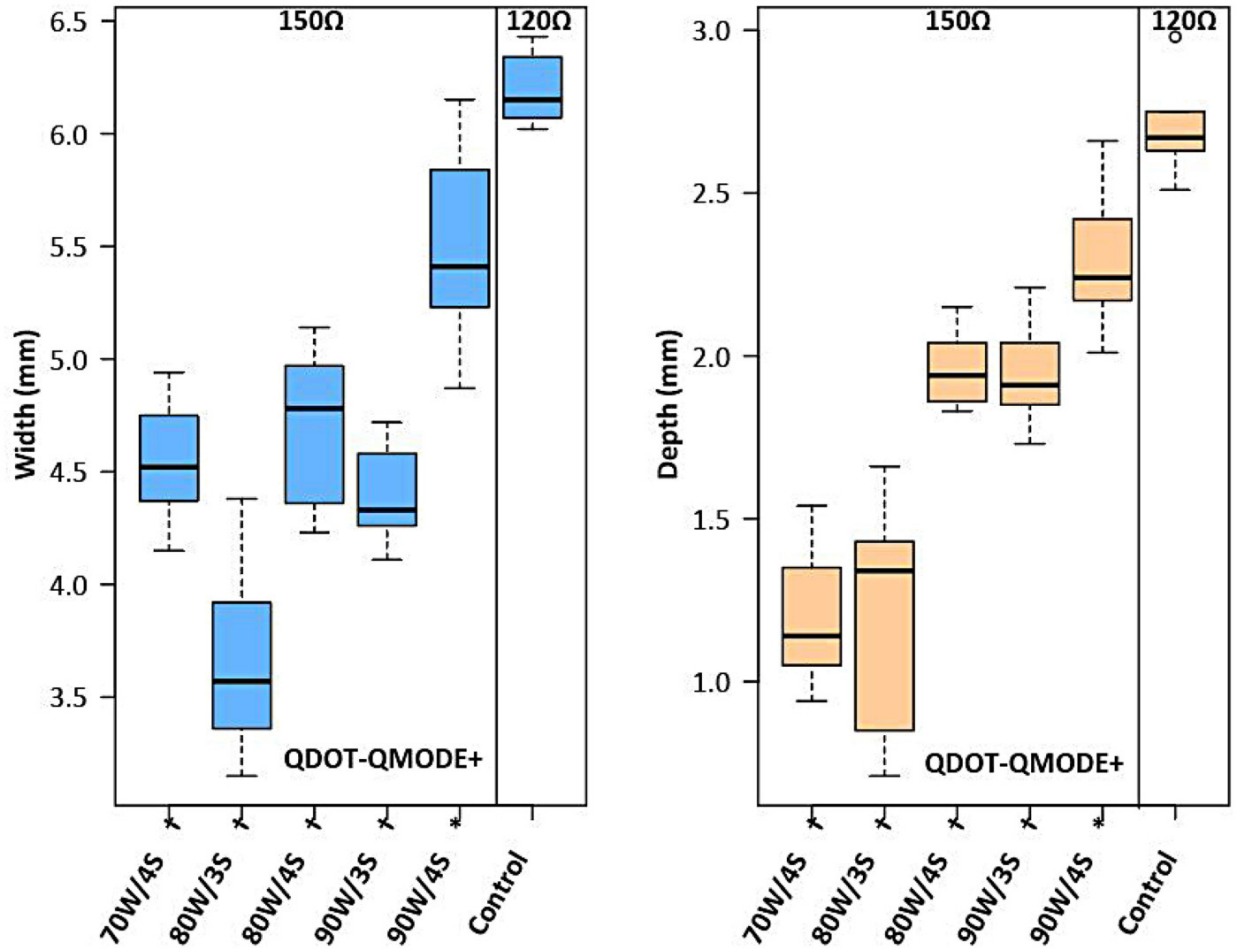
Box plots showing lesion width and depth for each tested ablation setting in 150Ω under QMODE+ ablation mode, compared to the control group (vHPSD-120Ω). Statistical significance is indicated as follows: *P < 0.05, #*P* < 0.01, †*P* < 0.001.

**Figure 10:**
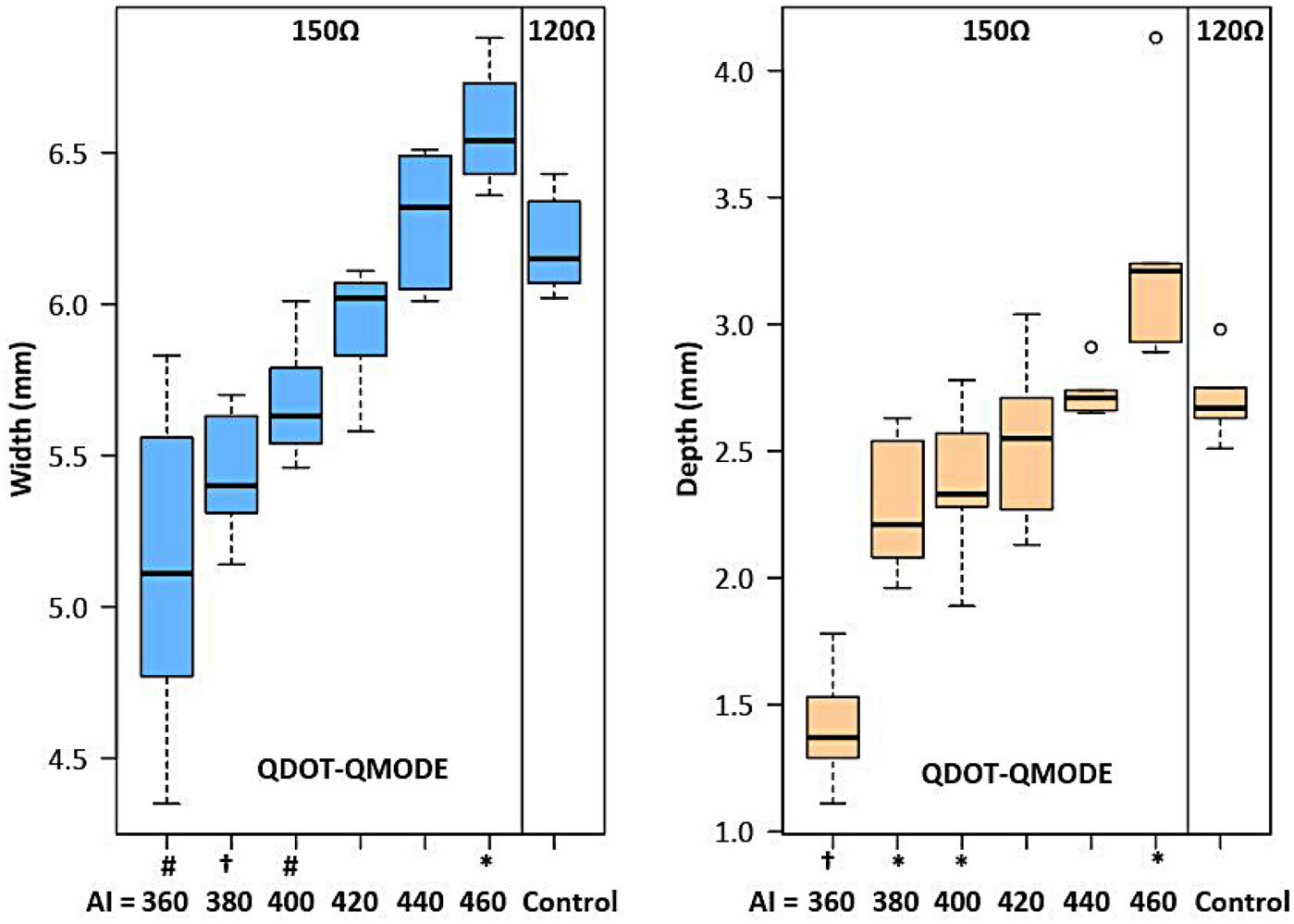
Box plot showing the damage width and depth for each tested ablation setting in QMODE ablation mode at 150Ω, compared to the control group (120Ω). Statistical significance is indicated as follows: *P < 0.05, #*P* < 0.01, †*P* < 0.001.

This study hypothesized how ablation settings should be adjusted to achieve comparable ablation lesions in QMODE+ ablation mode when impedances varied. Impedances were divided into three groups: 90Ω, 120Ω, and 150Ω, with the 120Ω group (90 W, 4 s) serving as the control (intermediate impedance). Figure 8 showed that in the 90Ω group, the parameters 80 W/3 s, 80 W/4 s, and 90 W/3 s yielded lesions comparable to those in the control group, with no statistical differences in depth or width (Supplementary Table S1). In contrast, Figure 9 indicated that lesion depths and widths in the 150Ω group were smaller than those in the control group (Supplementary Table S2). To ensure consistent ablation damage at 150Ω, we conducted an additional set of experiments using QMODE mode at 150Ω, comparing with those in the control group. Results showed that at 150Ω with an AI of 400/420 in QMODE mode, lesion surface width and depth were similar to those in the control group (Figure 10 and Supplementary Table S3).

## 4 Discussion

### 4.1 Effects of different ablation parameters on damage degree in QMODE+ ablation mode

In this *ex vivo* experimental model, our primary aim was to compare lesions arising from different ablation parameters, all performed using the QDOT-MICRO catheter in temperature-controlled mode. Notable differences in lesion width and depth were observed. In power-temperature control mode, it is well established that higher CF correlates with greater tissue damage, attributed to increased catheter tip-tissue contact area and enhanced current flow into tissue (19). In this study, when CF exceeded 15 g under QMODE+ ablation mode, tissue temperature decreased more rapidly—particularly at 25 g—resulting in relatively smaller lesions. This phenomenon was attributed to more frequent power titrations inherent to temperature-control mode. The QDOT-MICRO catheter, equipped with three distal and three proximal thermocouples for monitoring tissue temperature throughout ablation, provides sensitive temperature tracking and dynamic power adjustment (20). This mechanism effectively balanced CF and RF power to achieve the observed lesion characteristics, as summarized in Table 1. Previous studies (21, 22) have reported that both power wattage and RF duration are associated with lesion sizes, with higher power and longer RF duration correlating with increased lesion dimensions. Intermediate power settings (51–80 W for 4 s) were included to characterize the dose–response relationship between RF power and lesion characteristics in QMODE+ ablation mode, rather than to replicate routine clinical ablation strategies.

Consistent with previous reports (23), this study confirmed that perpendicular contact achieves greater ablation tissue depth than parallel contact. For ablation procedures: parallel contact was preferred at low impedance (to reduce lesion depth and avoid over-damage) and perpendicular contact at high impedance (to ensure therapeutic depth); prioritize parallel contact for fragile sites (e.g., thin posterior walls) to improve safety. This strategy balances ablation efficacy and procedural safety.

Bortone et al. (24) used CARTO and QDOT-MICRO catheters in temperature-controlled mode in eight adult sheep, confirming that lesions with interlesion distances of 3, 4, 5, and 6 mm were histologically transmural and continuous. In our experiment, interlesion distances of 3 mm and 4 mm under QMODE+ ablation mode significantly increased interlesion depth, with a corresponding augmentation in the extent of interlesional damage; therefore, in thinner regions such as the posterior wall of left atrial, these ranges of interlesion distance might increase atrioesophageal fistula risk due to proximity to the esophagus. For the atrial anterior wall—where myocardial tissue is relatively thicker and less susceptible to transmural injury—an interlesion distance of 3–4 mm can be adopted to enhance the continuity and extent of ablation lesions, thereby ensuring sufficient damage. In contrast, a 6 mm interlesion distance resulted in insufficient depth at the lesion junction, with gaps between the ablation points impairing continuity, which may increase the recurrence risk. As shown in Figure 6, lesions were discontinuous obviously when interlesion distance was ≥7 mm. Given that each condition included only five lesions, 5 mm can be considered a practical reference interlesion distance within the tested *ex vivo* conditions. Its applicability should be adjusted based on myocardial wall thickness and anatomical location.

The lower the impedance, the greater the damage. In our study, adjusting the RF duration exerted a significant impact on lesion characteristics, with substantial differences in lesion depth and width observed per unit change in RF time (Figure 4B). Therefore, to fine-tune the ablation lesion while ensuring procedural safety, we prefer to adjust the power output rather than modifying the RF application time. To investigate effects of different impedances on lesions, TTC staining was used to characterize the ablation damage. At 90Ω, the lesions were the most severe, with coagulative necrosis observed in the center—a finding not noted at other impedance values. Specifically, the 90Ω lesions were clearly demarcated into three distinct regions: (1) a central dark brown zone, (2) a lighter peripheral zone, and (3) normal red myocardial tissue.

### 4.2 Ablation performance and parameter adjustments across different impedances

When the same power was applied at different impedances, the depth and width of ablated lesions varied significantly. Specifically, as impedance elevated, lesion size decreased; conversely, as impedance decreased, lesion size increased (25). At low impedance (90Ω), RF power output should be reduced. In QMODE+ ablation mode, our study found: (1) ablation parameters of 80 W/3 s, 80 W/4 s, and 90 W/3 s at lower impedance (90Ω) produced lesion characteristics comparable to those under medium impedance (120Ω); (2) lesions produced at higher impedance (150Ω) differed from those under medium impedance (120Ω). To ensure consistent ablation damage at 150Ω impedance, we added another set of experiments using QMODE, which found that lesions at AI = 400/420 (150Ω) were similar to those in medium impedance QMODE+ group (120Ω).

## 5 Clinical implications

The effects of different ablation parameters on cardiac tissue damage under QMODE+ ablation modes were analyzed. This analysis facilitates a better understanding of the ablation characteristics of new ablation modes, thereby guiding clinical procedures more effectively and safely. In particular, the study addresses how to adjust catheter ablation parameters based on varying impedances: energy wattage or ablation duration can be reduced at lower impedance under QMODE+ ablation mode, and switching to QMODE is recommended at higher impedance. Notably, impedance in this study was modified by adjusting saline concentration and dispersive electrode distance, which does not fully replicate clinical local impedance changes (influenced by tissue properties, catheter contact, and blood flow). Thus, these findings are specific to the experimental setting and require clinical validation.

## 6 Limitations

Like any preclinical experiment, this study was conducted using *in vitro* models, whose biophysical properties may differ from those of humans and from clinical ablation procedures. The *ex vivo* experimental model lacked coronary perfusion and did not account for cardiac or respiratory movements. Additionally, a salt solution was used instead of blood in the tissue bath to facilitate direct visualization of catheter-tissue contact and ensure accurate targeting of ablation sites. The differences in conductivity between the salt solution and blood may have influenced ablation lesion formation. Additional limitations include: (1) Small sample size (5 lesions per group), which limits statistical power for detecting subtle differences, especially for 1 mm increment interlesion distance comparisons. (2) Incomplete lesion geometry assessment: RF lesions are typically elliptical, but this study only measured surface width and maximum depth, without quantifying area, volume, or junctional continuity. This restricts comprehensive evaluation of interlesion gap formation.

## 7 Conclusions

Routine QMODE+ ablation with 90 W power, 4 s RF duration, catheter CF <25 g, 5 mm interlesion distance (reference in this *ex vivo* model), and perpendicular contact achieves optimal lesion formation. To ensure consistent damage across different impedances, adjust QMODE+ parameters at low impedance and switch to QMODE at high impedance. Parallel contact could be preferred at fragile sites or under low impedance to reduce lesion depth and avoid over-damage.

## Data Availability

The datasets presented in this study can be found in online repositories. The names of the repository/repositories and accession number(s) can be found in the article/Supplementary Material.
