# Supplementary material for: QMODE+ ablation mode: optimal parameter setting and impedance-adapted strategies
Source: Front Cardiovasc Med. 2026 Feb 5;13:1743828. doi: 10.3389/fcvm.2026.1743828 (PMC12916685; doi:10.3389/fcvm.2026.1743828)
Supplement: Supplementary file 1 [file Datasheet1.pdf]

Supplementary Table S1: Lesions characteristics under different ablation parameters in 90Ω

Lesion Parameters	70W/3S	70W/4S	80W/3S	80W/4S	90W/3S	90W/4S	Control (120Ω, 90W/4s)
	N=5	N=5	N=5	N=5	N=5	N=5	N=5
Surface width	5.25±0.67	5.51±0.50	5.92±0.73	6.44±0.37	6.43±0.20	6.97±0.57	6.20±0.18
Lesion depth	1.82±0.44	2.16±0.17	2.51±0.23	2.92±0.22	2.81±0.39	3.18±0.38	2.71±0.17
p-value VS control group							
Surface width	0.016	0.020	0.428	0.216	0.089	0.019	\
Lesion depth	0.003	0.011	0.163	0.138	0.578	0.036	

Supplementary Table S2: Lesions characteristics under different ablation parameters in 150Ω

Lesion Parameters	70W/3S	70W/4S	80W/3S	80W/4S	90W/3S	90W/4S	Control (120Ω, 90W/4s)
	N=5	N=5	N=5	N=5	N=5	N=5	N=5
Surface width		4.55±0.31	3.68±0.49	4.69±0.39	4.40±0.25	5.50±0.23	6.20±0.18
Lesion depth		1.20±0.24	1.20±0.40	1.96±0.13	1.95±0.18	2.30±0.25	2.71±0.17
p-value VS control group	\						
Surface width		<0.001	<0.001	<0.001	<0.001	0.019	\
Lesion depth		<0.001	<0.001	<0.001	<0.001	0.017	

Supplementary Table S3: Lesion characteristics under different ablation parameters in QMODE ablation mode in 150Ω

Lesion Parameters	AI=360	AI=380	AI=400	AI=420	AI=440	AI=460	Control (120Ω, 90W/4s)
	N=5	N=5	N=5	N=5	N=5	N=5	N=5
Surface width	5.12±0.59	5.43±0.23	5.69±0.21	5.92±0.22	6.28±±0.24	6.60±0.21	6.20±0.18
Lesion depth	1.42±0.25	2.28±0.29	2.30±0.34	2.54±0.36	2.73±0.11	3.28±0.50	2.71±0.17
p-value VS control group							
Surface width	0.005	<0.001	0.003	0.059	0.590	0.015	\
Lesion depth	<0.001	0.023	0.040	0.376	0.783	0.042	
